# Economic Evaluation of Interventions for Treatment of Neonatal Opioid Withdrawal Syndrome: A Review

**DOI:** 10.3390/children8070534

**Published:** 2021-06-23

**Authors:** Evelyn Lee, Deborah Schofield, Syeda Ishra Azim, Ju Lee Oei

**Affiliations:** 1 Centre for Economic Impacts of Genomic Medicine, Macquarie University, North Ryde, NSW 2109, Australia; deborah.schofield@mq.edu.au; 2 School of Women’s and Children’s Health, University of New South Wales, Kensington, NSW 2031, Australia; syeda_ishra.azim@unsw.edu.au (S.I.A.); j.oei@unsw.edu.au (J.L.O.)

**Keywords:** economic evaluation, cost-effectiveness, neonatal withdrawal syndrome, neonatal abstinence syndrome, neonatal opioid withdrawal syndrome, opioid exposed, maternal substance use

## Abstract

This study assessed the economic evidence on the pharmacological and non-pharmacological management of infants with neonatal opioid withdrawal syndrome (NOWS). Six databases were searched up to October 2020 for peer-reviewed studies. After titles and abstracts were screened, 79 studies remained for full-text review, and finally, 8 studies were eligible for inclusion in the review. The methodological quality of included studies was assessed using the Drummond checklist. The review showed significant limitations in these studies, with one study being rated as good and the remaining seven studies as of poor quality. There are methodological issues that require addressing, including a lack of detail on cost categories, a robust investigation of uncertainty, and extending the time horizon to consider longer-term outcomes beyond the initial birth hospitalization. Despite these limitations, existing evidence suggests non-pharmacological strategies such as rooming-in were associated with a shorter hospital stay and a decreased need for pharmacological treatment, thereby lowering hospitalization costs. The review highlights the paucity of high-quality studies assessing the cost-effectiveness of intervention strategies for NOWS. There is also a lack of evidence on long-term outcomes associated with NOWS and the treatment of NOWS. The inclusion of economic analyses in future studies will provide evidence to inform policymakers on resource allocation decisions for this patient population.

## 1. Introduction

Substance use during pregnancy is a significant and growing global public health concern. In the United States (US), illicit drug use (specifically opioid use disorder) among pregnant women has increased almost four-fold between 2004 and 2012, with at least one infant born with signs of withdrawal or neonatal opioid withdrawal syndrome (NOWS), formerly known as neonatal abstinence syndrome (NAS), every 15 min [1,2,3]. Similar trends in the rate of NOWS have also been reported in other countries, including the United Kingdom and Canada [4,5,6].

Neonatal opioid withdrawal syndrome is an opioid withdrawal syndrome experienced by substance-exposed infants shortly after birth. However, other non-opioid substances may reproduce neuro-behavioral dysregulation, consistent with an abstinence/withdrawal syndrome [7,8,9]. As a result, most infants diagnosed with NOWS or showing signs of withdrawal often require pharmacological treatment for withdrawal, resulting in prolonged hospital stays and neonatal intensive care unit admissions. 

The economic burden of NOWS, especially to the healthcare system, is considerable. For example, in 2014, the estimated costs of birth admission associated with NOWS were USD 500 million for the US, and USD 21.5 million for Canada (CAD 26.9 million) [10,11,12]. Although the long-term outcomes of children after NOWS have not been well studied, opioid exposure is associated with poor neurodevelopmental outcomes, with potential long-term effects including poor educational achievement and work productivity [13]; a previous study estimated a lifetime cost exceeding USD 100,000 per year per child [14].

With rising healthcare costs and limited resources, significant efforts have been made to identify effective treatment strategies to reduce pharmacotherapy and the length of hospital stay for infants with NOWS. Currently, pharmacological treatment such as buprenorphine is the mainstay of treatment for NOWS. However, recent evidence suggests other newer management approaches, such as quality improvement initiatives that standardize structure and treatment protocols, and non-pharmacological factors (e.g., breastfeeding, parental presence, and rooming-in), reduced the length of hospital stay and decreased the need for pharmacologic treatment, thereby lower hospitalization costs. 

As different management of NOWS affects outcomes such as the length of stay, and consequently the hospitalization costs, economic evaluation provides an important evidence base for policymakers when making resource allocation decisions [15].

This review aims to synthesize the literature on the cost-effectiveness of interventions for NOWS and uses the Drummond checklist to assess the methodological quality of the included studies. To our knowledge, there has not previously been a review of the economic evidence of interventions for the treatment of NOWS.

## 2. Materials and Methods

This review was conducted and reported following the preferred reporting items for systematic reviews and meta-analyses (PRISMA) guidelines [16]. 

### 2.1. Search Strategy and Data Sources

The “PICO” statement was used to define the search criteria for the review, and identify the patient population’s specifics, intervention/comparator, and outcomes. The electronic databases, Medline (Ovid), Embase (Ovid), CINAHL (EBSCOhost), EconLit (EBSCOhost), Web of Science and the NHS Economic Evaluation Database (NHS EED), were searched for the literature of full economic evaluations of treatment for NOWS. The MEDLINE, Embase, CINAHL, and Web of Science databases were selected, as they have strong coverage of medicine, biomedicine, pharmacy, health and drug literature. EconLIT and the NHS EED provide coverage of economics literature and economic evaluations of health technologies, respectively. Gray literature was not included, as most economic evaluations are published or cited in scientific or economic journals, which would be identified during the extensive online literature search. The present review was not registered in any database.

We restricted our search to full-text studies published in English between January 2000 and October 2020. The search strategy included terms such as “neonatal abstinence syndrome” or “substance withdrawal syndrome”, and “economic evaluation” or “cost analysis” (see Supplementary Materials File S1 for full search strings). We also carried out a manual search based on references found in the reviewed articles. 

### 2.2. Data Extraction 

The data extraction and quality assessment were conducted by two authors (E.L. and S.I.A.) who independently screened titles and abstracts according to PICOS criteria for inclusion and exclusion, as specified in Table 1. Disagreement between reviewers was resolved through discussion, and in case of disagreement, issues were resolved by consensus. 

The main characteristics of the studies identified were populated onto a data extraction template that included country, patient population, intervention and comparator, type of interventions, time horizon, and key findings and conclusions. The primary outcome measures were clinical and economic data relating to the cost-effectiveness analysis. Due to the substantial methodological and statistical heterogeneity across the studies, a narrative synthesis of data was conducted without meta-analysis. 

To facilitate comparison, identified cost estimates were converted and inflated to 2020 US dollars (USD) by using the consumer price index [17]. The exchange rate on June 30 of the year that the study was conducted was used to convert other currencies to USD [18]. The costs of interventions for the treatment of NOWS were presented according to the types of intervention.

### 2.3. Study Quality Assessment

The Drummond checklist was used for the critical appraisal of the studies [15]. This 10-item checklist covers the following: (1) study design—whether the objectives of the study were reported and the economic perspective (i.e., who bears the costs) was reported and justified; (2) data collection—whether the resource use and unit cost data collection was reported; and (3) analysis and interpretation of results—how results were analyzed and interpreted. Each item in the checklist is assigned a score of 1 that fulfills the Drummond criteria, and subcategories are given equal weight. For example, items that are classified into two subcategories are given an equal weight of 0.50. Although the checklist is not a scoring instrument, all identified papers were divided into three quality categories according to the proportion of items achieved: good (8–10), average (4–7), and poor (<4), as has been classified in other review studies adopting the same checklist [19].

## 3. Results

Overall, our search identified 293 potentially relevant studies from the database search, hand-search, and cross-references. After title and abstract screening and the removal of duplicates, the resulting 79 articles were obtained for full-text review. Finally, eight studies were included in the review. The selection process, according to PRISMA, is shown in Supplementary Materials Figure S1.

### 3.1. Study Characteristics 

Table 2 summarizes the characteristics of studies that met the inclusion criteria [20,21,22,23,24,25,26,27]. These studies compared the effectiveness and cost-effectiveness of interventions to prevent the need for newborn withdrawal medication and reduce the severity of withdrawal. No studies examined the effects of medication and models of care on non-opioid exposure, including methamphetamine, cannabis, cocaine and prescription medications. The types of interventions reported in the studies include the rooming-in model of care, inpatient–outpatient management, and assessment methods. The study population sizes ranged from 77 to 287 children diagnosed with NOWS. 

All but one study focused primarily on care delivered in a hospital setting, and the clinical outcomes and costs associated with interventions were derived from hospital records. One study used a decision-analytic model populated with secondary data sources to assess the cost-effectiveness of interventions for treating NOWS [23]. 

All the studies reported an economic evaluation of an intervention for NOWS treatment, comparing them with standard care using a historical control group [20,21,22,24,25,26,27]. One model-based study was identified where the effects were expressed in terms of quality-adjusted life-years (QALY) [23]. The majority of the studies were cost–consequence analyses, where costs and effects were not aggregated into a single measure but were instead expressed in terms of mean cost per patient. 

All observational cohort studies were based on inpatient period (median or mean length of stay) [20,21,22,24,25,26,27]. Avram and colleagues [23] adopted a lifetime horizon to estimate the incremental cost per QALY of rooming-in for infants with NOWS. Costs and QALYs were discounted at 3% per year. In the study, the “lifetime horizon” refers to the follow-up period to capture the downstream costs (or cost saving) and benefit. The majority of studies were conducted in the United States (six studies) [20,21,22,23,24,25]; one study originated in Canada [26] and another from Germany [27].

### 3.2. Type of Interventions

#### 3.2.1. Rooming-In Model of Care

Two studies [23,27] assessed the outcomes of rooming-in as the initial management approach for infants with NOWS. Although they differed in study design and choice of outcome measures, both studies concluded that rooming-in was associated with a lower cost. 

Avram and colleagues used a decision-analytic model to assess the lifetime cost-effectiveness of rooming-in compared with no rooming, with reported cost-savings of USD 521 million, and 12,333 additional maternal and neonatal QALYs for the rooming-in model of care. The rooming-in approach was also found to have a 92.4% probability of being cost-effective against a willingness-to-pay threshold of USD 100,000 per QALY [23].

Another study found that rooming-in was associated with lower healthcare costs of USD 6978 per infant, as a result of reduced hospital stays from 41.5 to 33 days, and a decreased need for pharmacological intervention by 17% from a healthcare perspective [27]. 

#### 3.2.2. Withdrawal Assessments

The proportion of infants medicated for withdrawal may be determined by the type of assessment methods used to determine withdrawal severity. A total of five studies [20,21,22,24,25] compared the outcomes of the most widely used assessment methods, from the Finnegan neonatal abstinence severity score to quality improvement initiatives that standardized treatment protocols, the assessment approach [e.g., eat, sleep and console (ESC), and a greater emphasis on non-pharmacological factors (e.g., family-centered communication) for managing NOWS. All studies consistently found quality improvement initiatives were associated with cost savings as a result of shorter hospitalizations and a decreased need for pharmacological intervention. 

For example, Wachman and colleagues assessed the outcomes of a “Plan–Do–Study–Act” quality improvement project that emphasized non-pharmacological approaches, such as parental presence. They found a significantly shorter hospital stay from 17.4 days to 11.6 days, and fewer pharmacological interventions from 87.1% to 40%, thereby reducing average hospitalization charges by USD 11,667 per affected infant [22].

#### 3.2.3. Inpatient versus Outpatient Pharmacologic Weaning Model of Care 

Kelly and colleagues compared hospital-based versus outpatient weaning and found that at-home oral morphine weaning may offer several potential advantages, including a slower morphine wean and increased mother-infant bonding time, thereby decreasing hospitalization costs [26].

### 3.3. Economic Analysis Characteristics 

Descriptive characteristics of the economic evaluation of the included studies were reported in Table 2. All studies reported direct medical costs, such as hospitalization costs, but did not provide detailed information on specific cost components (e.g., specialist’s fee, medication, diagnostics) in their economic evaluations. One study took a societal perspective by considering the lifetime cost of care of a neonate with neurodevelopmental impairment due to NOWS [23]. In the remaining seven studies, this perspective was not mentioned, although a healthcare perspective could be inferred. 

#### 3.3.1. Adjustments for the Timing of Costs and Benefits

The time horizon for most studies in this review was reported based on an initial hospital stay of less than a year, and therefore discounting was not applied. Avram and colleagues discounted the total lifetime costs of a person with neurodevelopment impairment due to NOWS by applying a 3% discount rate to both the costs and outcomes. It is acknowledged that a lifetime horizon can be difficult to model because of the absence of long-term efficacy data.

#### 3.3.2. Uncertainty of Estimates 

Uncertainty was considered in most studies, either through sensitivity analyses in the decision-analytic models and statistical tests (e.g., chi-square) in the retrospective cohort studies. Avram and colleagues assessed uncertainty using one-way sensitivity analysis and probability sensitivity analysis (Monte Carlo simulations) [23]. 

### 3.4. Quality Assessment of Economic Methods 

The Drummond checklist was applied to the eight studies, with one being rated as good and the remaining studies as being of poor quality. The seven studies assessed as being of poor quality were cost consequence analysis studies, where the units of outcome were expressed but little detail on direct cost categories was provided. In these studies, the incremental costs and outcomes of alternatives were also not reported. 

**Table 2:** Characteristics of included studies.

First Author; Year; Country	Infants with NAS Sample Size (N)	Study Design; Types of Intervention; Time Horizon	Primary Measure of Outcome	Comparator	Perspective; Type of Costs	Key Findings/Conclusion	Quality Ratings
Grossman, 2017; United States [20]	287	Retrospective chart review; quality improvement initiatives; birth to end of initial hospital stay	Length of stay, need for pharmacologic treatment and hospitalization cost	Finnegan neonatal abstinence scoring system, direct admission to NICU	Healthcare; direct medical costs	Compared to standard care in the pre-intervention period, QI intervention resulted in a shorter duration of hospital stay (22.4 versus 5.9 days) and lower average costs of hospitalization (USD 47,944 versus USD 11,005) for infants with NAS. ICER not reported.	Poor
Holmes; 2016; United States [21]	163	Retrospective chart review; quality improvement initiatives; birth to end of initial hospital stay	Need for pharmacologic treatment, length of stay and hospitalization cost	Rooming-in on the mother-infant unit and a minimum of 96 h observation period; pharmacological treatment and admission to NICU	Healthcare; Direct medical costs	Compared to standard care during the pre-intervention period, QI intervention resulted in a lower cumulative morphine exposure per infant, shorter duration of stay (16.9 versus 12.3 days) and average cost of hospitalization (USD 21,111 versus USD 9364) for infants with NAS (*p*-value not reported). ICER not reported.	Poor
Wachman; 2018; United States [22]	186	Retrospective chart review; quality improvement initiatives; birth to end of initial hospital stay	Length of stay. Secondary outcomes: need for pharmacologic treatment, breastfeeding initiation, and associated hospitalization charges	Finnegan assessment tool	Healthcare; direct hospital charges	Compared to standard care during the pre-intervention period, QI intervention significantly reduced the proportion of pharmacologically treated infants, duration of stay (17.5 versus 11.6 days) and average hospital charges from USD 33,280 to USD 21,613). No ICER was reported	Poor
Avram; 2020; United States [23]	23,200 hypothetical cohort	Decision model; rooming-in; lifetime projection	Maternal and child quality-adjusted life-years (QALY)	Not rooming-in	Societal; direct healthcare costs; lifetime cost of the neonate with neurodevelopmental impairment	Rooming-in and breastfeeding is the dominant strategy (i.e., less costly and with higher QALYs) resulting in a cost-saving of USD 521 million and an additional 12,333 QALYs Based on a cost-effectiveness threshold of USD 100,000 per QALY, the rooming-in and breastfeeding model was cost-effective. Sensitivity analysis showed that rooming-in was cost-effective in 94.2% of the simulations at a willingness-to-pay threshold of USD 100,000 per QALY	Good
Achilles; 2019; United States [24]	181	Prospective, observational study; quality improvement initiatives; birth to end of initial hospital stay	Need for medication and amount of dosage Secondary outcomes: length of stay and cost per affected infant	Finnegan neonatal abstinence scoring system and medication-weaning protocol	Healthcare; direct medical costs	Compared to standard care in the pre-intervention period, QI intervention resulted in infants requiring a lower cumulative dose of methadone exposure (*p* < 0.0001), and had a shorter duration of hospital stay (18.7 days vs. 10.9 days) and a lower average cost of hospitalization (USD 15,827 to USD 12,562). There was no significant difference in the average direct costs between the two groups. No ICER was reported	Poor
Devlin; 2017; United States [25]	190	Retrospective chart review; modified protocol with morphine dosing every 3 h and treated with clonidine; birth to end of initial hospital stay	Need for pharmacologic treatment, length of stay and associated hospitalization cost	Protocol that provides morphine every 4 h and utilized phenobarbital as adjuvant therapy	Healthcare; direct hospital charges	Modified protocol significantly reduced the need for pharmacologic treatment (35 to 26.5 days) and duration of hospital stay (42 to 33 days) resulting in an average decrease in hospital charges from USD 104,521 to USD 29,264 per infant	Poor
Kelly; 2014; Canada [26]	80	Retrospective observational study; weaning at home; two years	Primary outcome: re-admission to hospital for NAS-related concerns Secondary outcome: hospitalization cost	In-hospital weaning	Healthcare; direct medical costs	Compared to hospital weaning, infants weaned at home had a significantly shorter hospital stay (22 versus 16 days) resulting in a cost-savings of USD 11,537 per neonate. Home weaning also resulted in a longer duration of treatment and the likelihood of requiring adjuvant treatment in the NICU. No difference in readmission rate of up to 2 years after initial hospitalization	Poor
Hünseler; 2013; Germany [27]	77	Retrospective cohort study, rooming-in model of care; birth to end of initial hospital stay	Need for pharmacological treatment, length of stay and associated hospitalization cost	Not rooming-in	Healthcare; direct medical costs	Compared to no rooming-in, maternal rooming-in reduced duration of therapy (32.5 vs. 27 days), and cost of hospitalization (USD 20,466 versus USD 13,488, *p* = 0.014). There was no difference in the hospital stay (33.0 vs. 41.5 days). ICER was not reported	Poor

**ICER**: incremental cost-effectiveness ratio; **QI**: quality improvement initiative; **QALY**: quality-adjusted life-years.

## 4. Discussion

Our review reveals a lack of studies evaluating the cost-effectiveness of how to assess (e.g., Finnegan scale or ESC model), how to treat (e.g., methadone, morphine, or buprenorphine and respective dose effectiveness) and where to treat (e.g., inpatient, a home-based weaning mother’s room, or NICU) infants with NOWS. This is an important consideration, as the different management of NOWS may have a significant impact on the outcomes, such as on the length of stay, and consequently on the hospitalization costs [28]. 

The costs of the long-term effect of NOWS and the types of interventions (pharmacotherapy and non-pharmacological) for the treatment of infants with NOWS were also not well studied. Most economic studies focused on short-term outcomes (e.g., the cost of hospital stays) and the burden on healthcare costs. However, infants with a history of NOWS are likely to be at risk of failing to thrive and developmental delay, and require additional long-term support, including healthcare and special education. This is shown in a population-based follow-up study, where children with a previous diagnosis of NOWS had a poorer and deteriorating academic performance as early as eight years old, and required multiple hospitalizations compared to children without NOWS [13]. It is acknowledged that there are challenges in following up children with a previous diagnosis of NOWS, due to confounding environmental and social factors associated with substance-using mothers [29]. However, a longer-term time horizon will allow the economic assessment of resources consumed as a result of the developmental outcomes of children with NOWS and after treatment for NOWS.

In recent years, quality improvement initiatives and non-pharmacological interventions have proliferated as first-line treatment for infants with NOWS in a hospital setting. Although existing evidence suggests non-pharmacological interventions for NOWS resulted in cost savings, due to shorter hospitalizations and a decreased need for pharmacological intervention, the relative contribution of an individual strategy within nonpharmacological intervention (e.g., rooming-in, parental presence) and environmental modifications (e.g., standardized structure and processes) were often not assessed as a stand-alone strategy. 

This is important, as assessing each intervention individually helps identify positive outcomes and provides economic evidence that infants with NOWS can be potentially managed outside of the NICU setting at a lower cost [30,31]. For example, studies have consistently found that outpatient/home weaning was associated with shorter hospital stays and a higher breastfeeding rate. However, it is not clear if this is the effect of bonding between mother and infant, or community help in the care of infants receiving outpatient weaning that have contributed to the improved breastfeeding outcomes [32]. 

The methodological quality of economic evaluation in this review was mixed with most of the studies, using retrospective inpatient/clinical data that were not collected specifically for economic evaluation. Ideally, economic evaluations should report the QALY to improve comparisons between studies and to allow decision-makers to compare across diseases [15,33]. Furthermore, no studies have assessed the economic evidence regarding pharmacologic interventions for NOWS.

Our review highlighted the paucity of evidence on the cost-effectiveness of interventions for NOWS. In this review, we devised a broad search strategy to capture a range of outcomes from a list of electronic databases. The use of PRISMA guidelines also added transparency and completeness to the research. As the findings were categorized into types of interventions, it allows readers to assess the cost and effects of an intervention at a given willingness-to-pay threshold. 

## 5. Conclusions

This study makes an important contribution in guiding future research on developing a robust study design to capture the full health effects and costs associated with NOWS. This evidence is important to inform policymakers on resource allocation decisions for this patient population.

## Figures and Tables

**Table 1:** PICO criteria for inclusion and exclusion of studies.

Parameters	Inclusion Criteria	Exclusion Criteria
Population	Infants diagnosed with NOWS	Infants with symptoms not related to NOWS
Intervention	Novel assessment methods; nonpharmacologic treatment focused.	
Comparator	Usual/standard care (e.g., existing tools such as the Finnegan scale)	
Outcomes	Clinical and economic outcomes (e.g., length of stay; quality-adjusted life years)	No or partial evaluation (i.e., either costs or clinical outcomes of interventions were reported)

PICO: patient, intervention, comparison outcome; NOWS: neonatal opioid withdrawal syndrome.

## Supplementary Materials

The following are available online at https://www.mdpi.com/article/10.3390/children8070534/s1, File S1: Literature review search terms; Figure S1: PRISMA flow diagram of search results.
