# Supplementary material for: Economic Evaluation of Interventions for Treatment of Neonatal Opioid Withdrawal Syndrome: A Review
Source: Children (Basel). 2021 Jun 23;8(7):534. doi: 10.3390/children8070534 (PMC8306925; doi:10.3390/children8070534)
Supplement: Supplementary file 1 [file children-08-00534-s001.zip › Children-1215220 Supplementary File S1-done.pdf]

children

MDPI

Supplementary Materials

File S1. Systematic literature review search terms.

Database: MEDLINE (through OVID), CINAHL, Embase, ECONLIT, NHS Economic Evaluation Database and Web of Science
Inclusive dates: 2000 to October 31, 2020
Medline
(neonatal abstinence* OR substance withdrawal syndrome OR ‘substance withdrawal) AND (neonatal OR infant OR newborn) AND ('cost* OR cost-analysis OR economic evaluat* OR economic model* OR cost-benefit OR cost-effective*' OR cost-utility OR cost-minimisation)
Embase
((‘neonatal abstinence syndrome’ OR ‘substance withdrawal syndrome’ OR ‘substance withdrawal’) AND (‘neonatal’ OR ‘infant’ OR ‘newborn’ OR ‘infant’) AND (‘opioid’ OR ‘cannabis’ OR ‘cannabinoids’ OR ‘benzodiazepines OR ‘amphetamine’ OR ‘buprenorphine’ OR ‘methadone’ OR gabapentin’ AND ('cost' OR ‘cost-analysis’ OR ‘economic evaluation’ OR ‘economic model’ OR ‘increment cost-effect’ OR ''economic' OR 'cost-benefit analysis' OR 'cost-effectiveness' OR 'cost-utility' OR ‘cost-minimisation’))
Web of science
(TS= (neonatal abstinence syndrome) AND TS= (cost OR cost-analysis OR economic-evaluation OR economic-modelling OR ‘incremental cost effectiveness’ OR cost-benefit analysis OR cost-effectiveness OR cost-utility OR cost-minimisation) AND TS= (opioid OR cannabis OR cannabinoids OR benzodiazepines OR amphetamine OR buprenorphine OR methadone OR gabapentin)
CINAHL
TX (neonatal abstinence syndrome or neonatal withdrawal syndrome or NAS or neonatal abstinence or neonatal withdrawal) AND TX (cost effectiveness or cost benefit or economics or cost management or economics or cost utility or cost ministration or economic model or incremental cost effectiveness) AND TX (opioid analgesics or opioids or opioid or cannabis or benzodiazepines or methadone or buprenorphine or gabapentin)
EconLIT NHS EED
TX (neonatal abstinence syndrome or neonatal withdrawal syndrome or nas) AND TX (economy or economic or economics or economic impact or financial impact) OR TX (cost effectiveness or cost benefit or economics or cost management or economics) AND TX opioid OR TX cannabis OR TX (methadone or buprenorphine)
