# Supplementary figures and images for: Economic Evaluation of Interventions for Treatment of Neonatal Opioid Withdrawal Syndrome: A Review
Source: Children (Basel). 2021 Jun 23;8(7):534. doi: 10.3390/children8070534 (PMC8306925; doi:10.3390/children8070534)

Supplementary Material

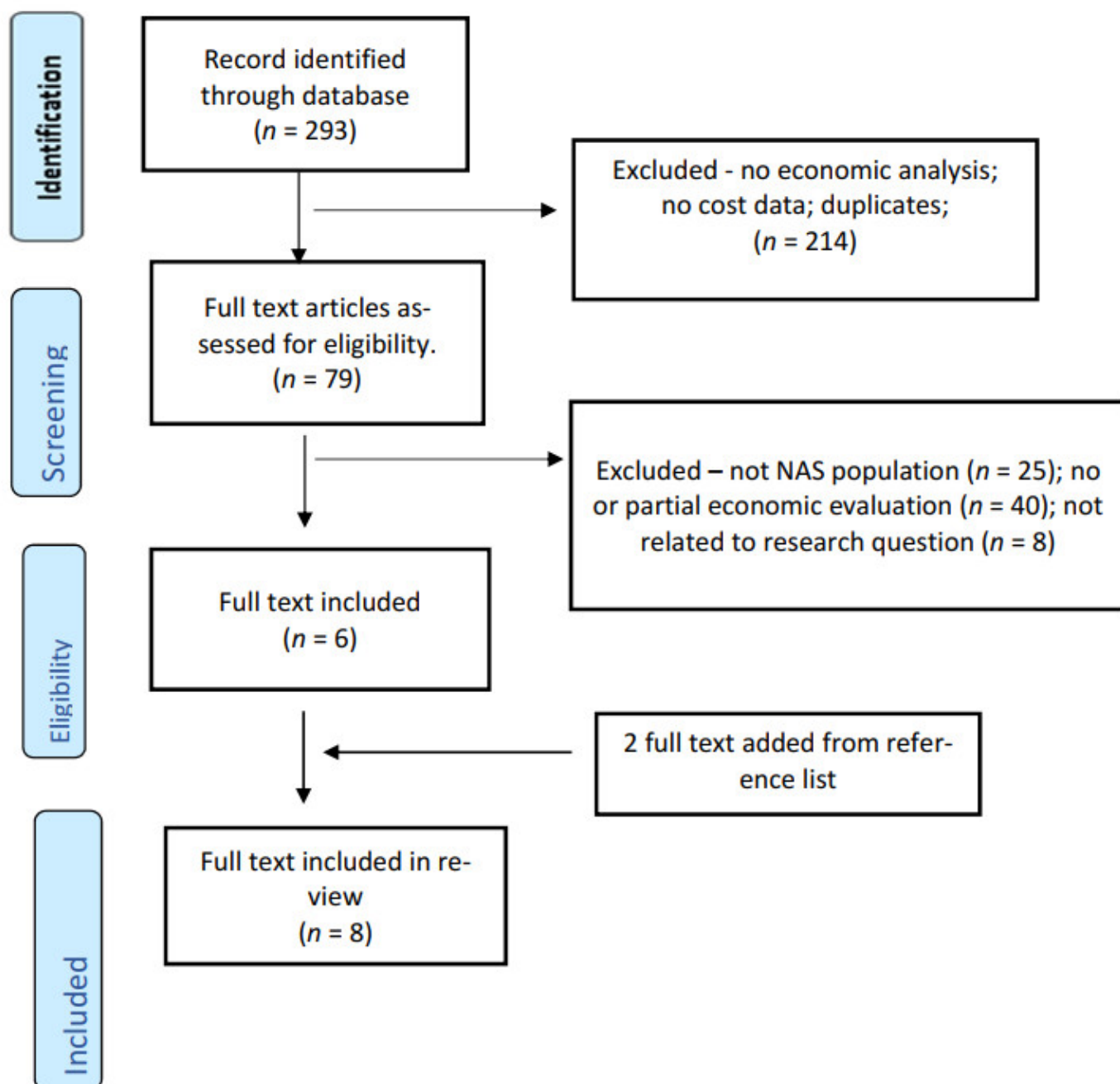

**Figure S1.** PRISMA Flow Diagram of search results.

Supplement: Supplementary file 1 [file children-08-00534-s001.zip › Children-1215220 Supplementary Figure S1-done.pdf]
